# Why we should not mistake accuracy of medical AI for efficiency

**DOI:** 10.1038/s41746-024-01047-2

**Published:** 2024-03-04

**Authors:** Karin Rolanda Jongsma, Martin Sand, Megan Milota

**Affiliations:** 1 Bioethics & Health Humanities, Julius Center, University Medical Center Utrecht, Utrecht University, PO Box 85500, 3508 CA Utrecht, The Netherlands; 2 TU Delft, Department of Values, Technology and Innovation, Faculty of Technology, Policy and Management, Jaffalaan 5, 2628 BX Delft, The Netherlands

**Keywords:** Medical ethics, Health policy

## Abstract

In the medical literature, promising results regarding accuracy of medical AI are presented as claims for its potential to increase efficiency. This elision of concepts is misleading and incorrect. First, the promise that AI will reduce human workload rests on a too narrow assessment of what constitutes workload in the first place. Human operators need new skills and deal with new responsibilities, these systems need an elaborate infrastructure and support system that all contribute to an increased amount of human work and short-term efficiency wins may become sources of long-term inefficiency. Second, for the realization of increased efficiency, the human-side of technology implementation is determinate. Human knowledge, competencies and trust can foster or undermine efficiency. We conclude that is important to remain conscious and critical about how we talk about expected benefits of AI, especially when referring to systemic changes based on single studies.

AI systems have proven to be accurate—in terms of positive predictive value (PPV) and sensitivity—for tasks that are time consuming or strenuous for health care professionals. Accuracy of those systems is important and a necessary condition for integrating AI in clinical practice. While it may seem natural to connect a technology’s accuracy with expectations about its efficiency, accuracy should not be mistaken for efficiency. Nevertheless, this consistently occurs in academic literature, policy reports and news items about AI. For example, when studies suggest that AI will reduce healthcare costs, resolve shortage of staff, optimize care in low resource settings, and even prevent burnout amongst health care professionals^1–8^, see Supplementary Table 1 for examples of these conflations. In some of these recent publications AI’s accuracy is thus mistakenly taken as a sufficient condition to achieve efficiency gains eg.^1,3,7,8^: In other academic papers, the accuracy of a technological system is even deemed equivalent to its efficiency ^4–6^. We consider this elision of concepts to be flawed and misleading.

First, the confusion of accuracy for efficiency in terms of workload reduction is flawed because it rests on a too narrow assessment of what constitutes workload in the first place. AI systems do not emerge out of thin air. A significant amount of human labor and time has been invested in the development and validation of these systems by data scientists, AI engineers and clinicians. Leaving this labor out of discussions about medical AI draws a too favorable a picture in terms of the total amount of human work needed. Furthermore, ongoing input and labor from medical professionals will be necessary, even after systems have been validated and integrated in the daily workflow. For example, AI systems for radiology and pathology will require a constant stream of expert-annotated images to maintain system accuracy^9^. If these annotations must be completed separately or differently from standard annotation processes, for example in a separate digital system, this additional labor will have to be factored into clinicians’ already heavy workload of clinical assessment, multidisciplinary deliberation, and patient communication. Importantly, health care professionals will likely need to maintain their ability to assess these images without the support of AI. This means that training and new responsibilities will come on top of their work schedules, thereby increasing their workload^10^. In addition, we must not forget that technology is imperfect^11^. AI systems will make mistakes, malfunction, or even breakdown. Mistakes can include biased outcomes, “hallucinations” and AI drift, which may seriously harm patients and therefore demand measures and increased awareness to counter these unwanted effects. This underscores the fact that complex technological systems such as medical AI can only function well when supported by an extensive and reliable technical infrastructure and the expertise of people like IT experts and data scientists. Substantial human labor will also be required by these experts to keep the systems up to date, to ensure that they continue to be accurate, and to monitor their proper functioning in the workplace^12^. Moreover, innovations that may seem like an efficiency win in the short-term may become sources of inefficiency in the long-term because of systemic changes, as we have learned from other technologies^13^. Emails, for example, enabled the rapid exchange of written text. But emails have not merely replaced letters, the new system also changed fundamentally what and how we communicate and thereby led to more frequent communication in the long run. People now spend more time writing and reading emails than they ever did on letters^14^. This should teach us that even if AI proves to be an accurate tool leading to efficiency gains in a narrow sense, other systemic shifts might nullify this efficiency gain. An increase in availability of accurate AI systems may, for example, result in institutional or policy recommendations to apply it more frequently or for multiple causes, which might eventually increase rather than decrease the workload of clinicians regardless of the presence of an AI support system.

Second, even if AI systems are accurate and experimental results support the claim of efficiency gain, we should not underestimate the influence of the human-side of technology implementation on such systemic effects. Health care professionals who operate these systems influence whether the possible benefits from the technology will be realized. Their knowledge and (technical) competencies can foster or undermine efficiency; even the most accurate AI system will be inefficient in the hands of a practitioner who is unable to use it correctly. Therefore, the potential benefits of technological systems can only be realized when used adequately in clinical practice and implemented under specific conditions. These conditions include the skills to handle such technologies and the willingness to bear new responsibilities^12^. Another major variable in this equation is the trust a health care practitioner will place in these systems. At least some minimal level of trust is needed to be willing to use an AI system in the first place. Trust is also an important factor when these systems are adopted in the clinical workflow, as it is generally argued that health care experts should stay in the loop e.g.^15^. More importantly, in their interactions with these new technologies, medical experts will have to critically consider when the advice of such systems should be followed in clinical decision-making and when it should be disregarded; in other words, when should health care experts trust and when should they distrust such systems? Given the computational power of medical AI, it can be reasonable for medical experts to follow the algorithm’s advice^16^. Yet, the academic literature indicates that putting too much trust in algorithms can be risky; clinicians may, for example, uncritically adopt an algorithm’s biased or wrong advice^17^. Too much trust in these systems may cause efficiency gains on the short term, but eventually cause more mistakes and, thus, patient harm and a loss of efficiency in the long run. In the other extreme, when health care professionals do not trust these systems at all and question the accuracy of such algorithms too much—as medical AI are typically prone to type 1 errors, or false-positives^18^ – this may result in a decrease of efficiency in clinical practice due to unnecessary additional tests.

We conclude that is important to remain conscious and critical about how we talk about expected benefits of AI in terms of accuracy and efficiency. First, we should refrain from drawing conclusions about systemic effects based on single studies. Hopes that technology will lead to increased efficiency are not unprecedented. However, historical research indicates that such hopes are only rarely, unequivocally fulfilled^10^. The systemic effects of these technologies can often only be assessed years after their introduction with the help of historians, philosophers of technology, sociologists, and empirical insights into the day-to-day experiences of users themselves^19^. In other words, we cannot be sure of the systemic effects before the technology is introduced to the clinic. Second, to do justice to the broader context and human labor involved in developing and deploying medical AI systems, it will be crucial to distinguish the benefits of AI more clearly in terms of effectiveness (getting more done) and efficiency (doing it with fewer resources)^10^. Explicitly distinguishing between these two dimensions in future research will help us ascertain whether additional support and work is necessary or whether fewer recourses are needed for the same or better results. Third, more research needs to be conducted on the relation between trust and efficiency: How does trust in these systems emerge and what are its consequences? Is the expectation of efficiency a cause of (unwarranted) trust in AI systems inducing the aforementioned problems of overreliance? Normative investigations that provide guidance into the reasonable grounds for trust (such as accuracy, efficiency and clinical value^16,20^) are important in and of themselves, but they will not necessarily result in widespread trust in these systems. For now, it remains to be seen whether accurate AI systems will lead to efficiency gains and workload reduction. In the meantime, we must proceed carefully and continue to critically assess whether emerging AI systems really fulfill the needs of clinical realities.

### Supplementary information

Supplemental File 1

## References

[1] Lång K (2023). Artificial intelligence-supported screen reading versus standard double reading in the Mammography Screening with Artificial Intelligence trial (MASAI): a clinical safety analysis of a randomised, controlled, non-inferiority, single-blinded, screening accuracy study. Lancet Oncol..

[2] van Leeuwen KG (2022). How does artificial intelligence in radiology improve efficiency and health outcomes?. Pediatr. Radiol..

[3] Pantanowitz L (2020). Accuracy and efficiency of an artificial intelligence tool when counting breast mitoses. Diagn. Pathol..

[4] Lin A (2021). Artificial intelligence: improving the efficiency of cardiovascular imaging. Expert Rev. Med. Dev..

[5] Lebovitz S, Levina N, Lifshitz-Assaf H (2021). Is Al ground truth really true? The dangers of training and evaluating ai tools based on experts' know-what. MIS Quart.

[6] Conant EF (2019). Improving accuracy and efficiency with concurrent use of artificial intelligence for digital breast tomosynthesis. Radiology: Artif. Intell..

[7] Granter SR (2017). AlphaGo, deep learning, and the future of the human microscopist. Arch. Pathol. Lab Med.

[8] Topol E. J. *Deep Medicine - How Artificial Intelligence Can Make Healthcare Human Again*. New York, Basic Books. (2019).

[9] Maloca PM (2019). Validation of automated artificial intelligence segmentation of optical coherence tomography images. PLoS ONE.

[10] Tenner, E. *The efficiency Paradox: What big data can’t do*. Knopf: New York (2018).

[11] Tenner, E. *Why Things Bite Back: New Technology and the Revenge Effect*. HarperCollins Publishers. (1996).

[12] Sand M, Durán JM, Jongsma KR (2021). Responsibility beyond design: Physicians’ requirements for ethical medical AI. Bioethics.

[13] Rochlin, G. *Trapped in the net: The unanticipated consequences of computerization*. Princeton University Press. (1998).

[14] Newport C. *A World Without Email: Reimagining Work in an Age of Communication Overload*. Penguin Random House: USA, pp. xvi (2021).

[15] Verghese A, Shah NH, Harrington RA (2018). What this computer needs is a physician: Humanism and artificial intelligence. JAMA.

[16] Durán JM, Jongsma KR (2021). Who is afraid of black box algorithms? On the epistemological and ethical basis of trust in medical AI. J. Med. Ethics.

[17] Kiani A (2020). Impact of a deep learning assistant on the histopathologic classification of liver cancer. NPJ Digital Med..

[18] Ihsan Fazal M, Ebrahim Patel M, Tye J, Gupta Y (2018). The past, present and future role of artificial intelligence in imaging. Eur. J. Radiol..

[19] Roest B, Milota M, Leget C (2021). Developing new ways to listen: the value of narrative approaches in empirical (bio)ethics. BMC Med. Ethics.

[20] London AJ (2019). Artificial intelligence and black-box medical decisions: Accuracy versus explainability. Hastings Cent. Rep..

