# Supplementary material for: Why we should not mistake accuracy of medical AI for efficiency
Source: NPJ Digit Med. 2024 Mar 4;7:57. doi: 10.1038/s41746-024-01047-2 (PMC10912629; doi:10.1038/s41746-024-01047-2)
Supplement: Supplementary file 1 — Supplemental File 1 [file 41746_2024_1047_MOESM1_ESM.docx]

| **Reference** | **Quote illustrating the conflation between efficiency and accuracy** |
| --- | --- |
| 1. Lång K, Josefsson V, Larsson AM, Larsson S, Högberg C, Sartor H, Hofvind S, Andersson I, Rosso A. Artificial intelligence-supported screen reading versus standard double reading in the Mammography Screening with Artificial Intelligence trial (MASAI): a clinical safety analysis of a randomised, controlled, non-inferiority, single-blinded, screening accuracy study. Lancet Oncol. 2023;24(8):936-944 | “ The expected benefits of the adoption of AI are to improve the screening efficiency by increasing the PPV, to decrease screening harms such as false positive results, and, ultimately, to reduce breast cancer mortality.”  “The results from this randomised trial support the findings of earlier retrospective studies, indicating a general potential of AI to improve screening efficacy and reduce workload.” |
| 2. van Leeuwen KG et al. How does artificial intelligence in radiology improve efficiency and health outcomes?. Pediatr Radiol 2022;52:2087–2093 | “AI-supported tuberculosis detection is especially useful in developing countries where staffing, expertise and financial resources are often limited. This can be used as an autonomous pre-screening tool to reduce the use of microbiological tests, which are more time-consuming and costly (levels 2, 3, 6) [10,11,12,13]. This is one of the first AI applications in radiology where the software functions autonomously and has taken over the task of the radiologist.” |
| 3. Pantanowitz L et al. Accuracy and efficiency of an artificial intelligence tool when counting breast mitoses. Diagn Pathol 2020;15,80 | “With regard to improved efficiency, the use of AI resulted in a 27.8% decrease in time for mitotic cell detection. In other words, for every 1 h spent searching for cells with mitotic figures without AI support, roughly 16.7 min could be saved using AI support.” |
| 4. Lin A et al. Artificial intelligence: improving the efficiency of cardiovascular imaging, Expert Review of Medical Devices 2020;17:6,565-577. | “To address these limitations, investigators have combined DL with atlas prior knowledge (27) and deformable models (28) to achieve accurate and efficient automated LV segmentation.”  “As detailed in this paper, applications of AI can greatly improve the efficiency of noninvasive cardiac imaging modalities, by facilitating image acquisition, automating the processes of image segmentation and quantification, and assisting physician interpretation.” |
| 5. Lebovitz S, Levina N. & Lifshitz-Assaf H. Is AI ground truth really true? The dangers of training and evaluating ai tools based on experts'know-what. MIS quarterly 2020;45(3). | “ Finally, managers decided that the benefits of moving forward with the Chest Triage tool outweighed its potential risks: “[The department chair] has already given his stamp of approval. I think as long as it’s efficient, there’s no questioning.” |
| 6. Conant, EF. et al. Improving accuracy and efficiency with concurrent use of artificial intelligence for digital breast tomosynthesis.Radiology: Artificial Intelligence 1.4 2019: e180096. | “The results of this study suggest that both improved efficiency and accuracy could be achieved in clinical practice by using an effective AI system.” |
| 7. Granter, SR., et al. "AlphaGo, Deep Learning, and the Future of the Human Microscopist." Arch Pathol Lab Med 2017;141(5): 619-621. | “Computers will increasingly become integrated into the pathology workflow when they can improve accuracy in answering questions that are difficult for pathologists. […] We predict that, over time, as computers gain more and more discriminatory abilities, they will reduce the amount of time it takes for a pathologist to render diagnosis, and, in the process, reduce the demand for pathologists as microscopists, potentially enabling pathologists to focus more cognitive resources on higher-lever diagnostic and consultative tasks […]” |
| 8. Topol, EJ. (2019). Deep Medicine - How Artificial Intelligence Can Make Healthcare Human Again. New York, Basic Books. | “[…]  we’ve seen the potential role of AI to change medicine by ameliorating the accuracy of diagnosis and making the process more efficient.” |
